# Comprehensive Characterization of Simple Sequence Repeats in Eggplant (*Solanum melongena* L.) Genome and Construction of a Web Resource

**DOI:** 10.3389/fpls.2018.00401

**Published:** 2018-03-28

**Authors:** Ezio Portis, Sergio Lanteri, Lorenzo Barchi, Flavio Portis, Luisa Valente, Laura Toppino, Giuseppe L. Rotino, Alberto Acquadro

**Affiliations:** ^1^Dipartimento di Scienze Agrarie, Forestali ed Alimentari – Plant Genetics and Breeding, Università degli Studi di Torino, Turin, Italy; ^2^Yebokey, Turin, Italy; ^3^CREA-GB, Research Centre for Genomics and Bioinformatics, Lodi, Italy

**Keywords:** database, eggplant, genome, microsatellite, *Solanum melongena*, SSR

## Abstract

We have characterized the simple sequence repeat (SSR) markers of the eggplant (*Solanum melongena*) using a recent high quality sequence of its whole genome. We found nearly 133,000 perfect SSRs, a density of 125.5 SSRs/Mbp, and also about 178,400 imperfect SSRs. Of the perfect SSRs, 15.6% were complex, with two stretches of repeats separated by an intervening block of <100 nt. Di- and trinucleotide SSRs accounted, respectively, for 43 and 37% of the total. The SSRs were classified according to their number of repeats and overall length, and were assigned to their linkage group. We found 2,449 of the perfect SSRs in 2,086 genes, with an overall density of 18.5 SSRs/Mbp across the gene space; 3,524 imperfect SSRs were present in 2,924 genes at a density of 26.7 SSRs/Mbp. Putative functions were assigned via ontology to genes containing at least one SSR. Using this data we developed an “Eggplant Microsatellite DataBase” (*EgMiDB*) which permits identification of SSR markers in terms of their location on the genome, type of repeat (perfect vs. imperfect), motif type, sequence, repeat number and genomic/gene context. It also suggests forward and reverse primers. We employed an *in silico* PCR analysis to validate these SSR markers, using as templates two CDS sets and three assembled transcriptomes obtained from diverse eggplant accessions.

## Introduction

The eggplant (*Solanum melongena* L., 2*n* = 2*x* = 24), also referred to as aubergine or brinjal, belongs to the Solanaceae family. After potato and tomato, eggplant represents the third most important solanaceous crop species and its bulk production is concentrated in China, India, Iran, Egypt and Turkey, with Italy and Spain representing the most important European Union producers^1^. Unlike tomato (*S. lycopersicum*) and potato (*S. tuberosum*), which belong to the *Potato* clade and are native to South America, eggplant belongs to the spiny Solanums (subgenus *Leptostemonum*) and it is native to the Old World. A minimum of two domestication events from the wild species *S. insanum* has been supported: one in India and one in southern China/SE Asia, with a possible additional and independent center of domestication in the Philippines (Meyer et al., 2012). Two other eggplant species are commonly grown in sub-Saharan Africa: the scarlet eggplant (*S. aethiopicum L.*) and the gboma eggplant (*S. macrocarpon L*). Both of them can be inter-crossed with S. melongena producing hybrids with intermediate fertility (Acquadro et al., 2017).

Advances in next-generation sequencing (NGS) platforms, through multiplexed sequencing of barcoded samples in a single run, have driven the costs down and considerably reduced the time needed for the whole genome sequencing of plant species. Genomes provide opportunities to develop a huge amount of novel molecular markers, which can be used for assessing the genetic variations within cultivars and germplasms, for the development of molecular genetic and physical maps, for identifying genes and quantitative trait loci controlling economically important traits and to assist breeding for crop improvement. Among them, microsatellites or simple sequence repeat (SSR) markers represent one of the most informative, versatile, and practical DNA-based markers used in plant breeding programs, since they are easy to score and have wide genomic distribution, codominant inheritance and a multi-allelic nature (Xu et al., 2013). The first sets of eggplant microsatellites was developed from the screening of small insert genomic libraries with di- and trinucleotide probes (Nunome et al., 2003a,b). Afterwards, Stàgel et al. (2008) developed a small set of SSR markers from genic DNA sequence lodged in public access databases, while Nunome et al. (2009) reported the identification of over 1,000 SSR markers from a screen of enriched gDNA and cDNA libraries. An extensive set of nearly 2,000 putative eggplant SSRs was later on isolated by Barchi et al. (2011) from RAD (restriction-site associated DNA) tags, of which a sub-set showed to be polymorphic among the parents of mapping populations. Additional informative SSR markers were also developed from *S. melongena* genomic libraries enriched for AG/CT motif (Vilanova et al., 2012) and, more recently, from transcriptome analysis of *S. incanum* and *S. aethiopicum*, two close relatives of the common eggplant (Gramazio et al., 2016). In eggplant SSR markers have been applied in diversity, phylogenetic as well as linkage mapping studies (Barchi et al., 2010, 2012; Munoz-Falcon et al., 2011; Fukuoka et al., 2012; Hurtado et al., 2012; Prohens et al., 2012; Cericola et al., 2013; Gramazio et al., 2017). Recently a high quality genome draft of eggplant, covering 1.06 Gb of gap-free sequence with an N50 of 2.9 Mb, was produced by a combination of Illumina sequencing and optical mapping and was anchored to 12 pseudomolecules ^2^ (The Eggplant Genome Consortium, 2017).

Here we report on the identification of single locus SSR markers in an eggplant genome-wide survey as well as on the development of a public dynamic microsatellite database ^3^, which represents a one-stop resource for the global community of scientists and breeders. The web resource provides user need-based primer designing facilities with mobile-friendly features, to facilitate rapid selection of suitable custom markers for a wide range of genetic analyses.

## Materials and Methods

### The SSR Content of the Eggplant Genome

The high quality genome of the eggplant inbred line “67/3,” recently sequenced (The Eggplant Genome Consortium, 2017) and available in the public domain at www.eggplantgenome.org, was downloaded in FASTA format. Its 12 pseudomolecules (representing partial sequences of each of the species’ chromosomes), along with all unmapped scaffolds, were chopped into manageable pieces using SciRoKo tool^4^. Perfect, compound, and imperfect SSRs were identified *in silico* using the SciRoKo SSR—search module (see footnote 4). A minimum of four repetitions together with a minimum length of 15 nt was requested. Any sequence was considered as a perfect SSR where a motif was repeated at least fifteen times (1 nt motif), eight times (2 nt), five times (3 nt) or four times (4–6 nt), allowing for only one mismatch. For compound repeats, the maximum default interruption (spacer) length was set at 100 bp. The coordinates (start/end position) of each SSR were matched with those of the gene space using Bedtools intersect^5^, using the default parameters with -loj (left outer join) option: where the overlap comprised at least 1 nt, the repeat was designated as a genic SSR. The putative function of genes carrying at least one SSR was defined by the GO (gene ontology) approach. Enrichment for GO terms was recognized by examining the set of genic SSRs upon the whole genome GO annotation dataset using the AgriGO suite^6^, collecting enriched GO terms with *P*-values < *e*-5 and a false discovery rate <0.01, and visualized through ReviGO ^7^.

### Collection of Genomic Sequences From Different Solanaceae Species

For comparison purposes, the draft genome sequence of the Asian eggplant cultivars ‘Nakate-Shinkuro’ (Hirakawa et al., 2014), together with the full genome sequences of 12 other plant species from the Solanaceae family (*Solanum lycopersicum, S. pimpinellifolium, S. pennellii, S. tuberosum, Capsicum annuum, C. chinense, C. baccatum, Nicotiana tabacum, N. attenuata, N. benthamiana, Petunia axillaris, P. inflata*) and their closely related species *Coffea canephora*, were collected from the related public database and scanned for the presence of perfect SSRs, using the same procedures described above. The source of each of the above full genome sequences is given in Supplementary Table S1.

### EgMiDB, an SSR Database for Eggplant

The Eggplant Microsatellite DataBase (*EgMiDB*) was developed to provide browsable access to the SSR data. This web application, based on a LAMP stack, comprises a client tier (client browser), a middle tier (Apache web server with PHP interpreter) and a database tier (MySQL DBMS). A user-friendly interface was developed using PHP, which is an open-source server-side scripting language. The set of *in silico* detected SSRs were stored in the MySQL database, using PHP scripts to parse the text file from SciRoKo. User need-based customized queries can be generated from the web interface and allow users to search the microsatellite marker information in MySQL database. A stand-alone version of Primer3^8^ is also provided to design primer pairs for any given SSR: its output lists alternative sets of primer pairs, and the characteristics of the expected amplicon.

### Marker Validation

An *in silico* validation was adopted to validate designed SSR primers. About 1000 SSR primer pairs, amplifying in genic regions, were randomly selected and tested with the primer search tool (version EMBOSS:6.6.0.0) in three transcriptomes (Yang et al., 2014; Ramesh et al., 2016; PRJNA247728, unpublished) and two CDS set from eggplant genomic projects published by Hirakawa et al. (2014) and The Eggplant Genome Consortium (2017). Custom scripts were used to count positive PCR results and ambiguous amplifications.

## Results and Discussion

### The SSR Content of the Eggplant Genome and Cross-Species Comparison

In the ∼1.06 Gb of the gap-free eggplant genomic sequence, we identified 132,831 perfect SSR motifs (125.5 SSR/Mb), which included 20,670 compound SSRs (**Table 1**). The number of imperfect SSR motifs was 178,407 (**Table 2**). The content and distribution of SSRs in the genome sequence of the eggplant breeding line “67/3” were compared with those of 14 other plant genomes, related to different degrees (25.3 Gb of sequence in total, about 2.2 million SSRs) and retrieved from databases (Supplementary Table S1). The number of perfect SSRs found in the “67/3” genome was more than one-third higher than the number detectable in another eggplant cultivar, “Nakate-Shinkuro” (**Table 1**). This indicates that the high quality of the recently released eggplant genome sequence markedly influenced the number of detectable microsatellites.

**Table 1:** A comparative survey of perfect SSRs across the 15 analyzed genome sequences.

Genome	Analyzed sequences (Mbp)	Perfect SSRs				Compound SSRs	
		Count	Density (SSRs/Mbp)	Cumulative (Mbp)	Cumulative (%)	Count	%
*Solanum melongena* inbred line 67/3	1058.6	132,831	125.5	3.40	0.32%	20,670	15.6%
*S. melongena* cv. Nakate-Shinkuro	833.1	84,152	101.0	2.30	0.28%	12,405	14.7%
*Solanum lycopersicum*	828.1	89,480	108.1	2.60	0.31%	24,815	27.7%
*Solanum pimpinellifolium*	688.9	54,829	79.6	1.30	0.19%	9,828	17.9%
*Solanum pennellii*	989.5	121,662	122.9	3.89	0.39%	48,229	39.6%
*Solanum tuberosum*	884.1	86,354	97.7	2.49	0.28%	13,690	15.9%
*Capsicum annuum*	3183.6	194,622	61.1	3.94	0.12%	16,119	8.3%
*Capsicum chinense*	2953.0	212,867	72.1	4.52	0.15%	23,601	11.1%
*Capsicum baccatum*	3194.8	277,513	86.9	7.04	0.22%	39,935	14.4%
*Nicotiana tabacum*	3729.1	266,145	71.4	6.86	0.18%	26,971	10.1%
*Nicotiana attenuata*	830.4	67,198	80.9	1.95	0.24%	8,829	13.1%
*Nicotiana benthamiana*	2969.8	239,598	80.7	6.34	0.21%	29,569	12.3%
*Petunia axillaris*	1259.2	156,839	124.6	4.48	0.36%	22,486	14.3%
*Petunia inflata*	1287.0	124,445	96.7	3.16	0.25%	14,473	11.6%
*Coffea canephora*	569.9	79,279	139.1	2.05	0.36%	8,096	10.2%

**Table 2:** Variation in repeat length among genomic eggplant perfect and imperfect SSRs.

SSR type	Perfect motif							Imperfect motif		
	Kinds	Count	%	Density (SSRs/Mbp)	Cumulative (Mbp)	Cumulative (%)	Mean repeat number	Count	%	Density (SSRs/Mbp)
Mono-	2	11,152	8.4%	10.5	0.21	6.1%	18.5	11,891	6.7%	11.2
Di-	4	56,880	42.8%	53.7	1.74	51.1%	15.3	62,513	35.0%	59.1
Tri-	10	49,088	37.0%	46.4	1.09	32.1%	7.4	49,873	28.0%	47.1
Tetra-	33	9,428	7.1%	8.9	0.19	5.5%	5.0	16,246	9.1%	15.3
Penta-	80	3,750	2.8%	3.5	0.09	2.8%	5.0	26,684	15.0%	25.2
Hexa-	207	2,533	1.9%	2.4	0.08	2.5%	5.5	11,200	6.3%	10.6
**Total/mean**	**336**	**132,831**	**100.0%**	**125.5**	**3.40**	**100.0%**	**11.4**	**178,407**	**100.0%**	**168.5**

The “67/3” eggplant genome was also found to include almost twice the number of perfect microsatellites compared to the wild species *S. pimpinellifolium* (54,829) and *N. attenuata* (67,198), but only half of those in *N. tabacum* (266,145) and *C. baccatum* (277,513). The cumulative length of the full collection of eggplant SSRs was 3.4 Mbp, which is 0.32% of the assembled genome, a percentage analogous to those of tomato (0.31%) and potato (0.28%), but considerably higher than found in *C. chinense* and *C. annuum* (0.15 and 0.12%, respectively). Compound SSRs represented 15.6% of the eggplant perfect SSRs, a proportion exceeded only in *S. lycopersicum* (27.7%) and *S. pennellii* (37.6%) (**Table 1**).

Considering all 14 species, genome size was found to be positively associated with the number of identified SSR motifs (*R*^2^ = 0.948, *P* < 0.01). However, as a general trend, species possessing larger genomes show lower SSR density (SSRs/Mb) (Morgante et al., 2002). This is the case for the three *Capsicum* species and for *N. tabacum* and *N. benthamiana*, but *S. pimpinellifolium* and *N. attenuata* are exceptions; their genome sizes are, respectively, 688.9 and 830.4 Mbp, but their microsatellite densities are comparable to those found in plant species with genomes three to five times larger.

However, although differences in genome size may contribute to the level of repetition of microsatellites, density of SSRs has been found not to be related to genome size (Zhao et al., 2012; Behura and Severson, 2014; Portis et al., 2016). The density of perfect microsatellites in the genome of the eggplant breeding line “67/3” is the highest observed within the Solanaceae family, although similar to those detected in *S. pennellii* and *P. axillaris*, and, in the context of this study, inferior only to that found in *Coffea canephora* (139.1 SSRs/Mb) (**Table 1**).

### Characterization of SSR Motifs by Different Length and Repeats

**FIGURE 1:**
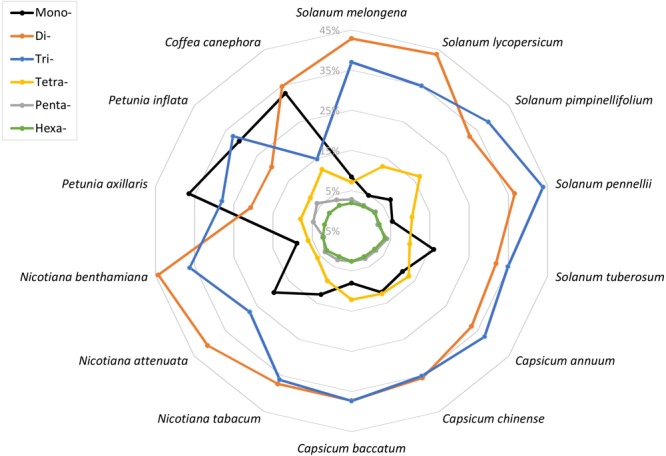
The representation of different simple sequence repeat (SSR) motifs across the 14 plant genomes.

Eggplant SSR motifs which predominated were the di- and trinucleotides (respectively 43 and 37% of all SSRs, with densities of 53.7 and 46.4 SSRs/Mbp), with lesser proportions of mono- (8%) and tetranucleotides (7%); the penta- and hexanucleotide repeats contributed <5% (**Table 2**). Dinucleotide sequences were in the majority, amounting to 1.7 Mbp (51.1% of the cumulative length of all SSR motifs). Dinucleotides were also the most common type in tomato and in *Nicotiana* species. Among the imperfect SSR motifs the occurrence of mono- to trinucleotide motifs was low compared to that seen among the group of perfect SSRs. There were relatively larger amounts of longer motifs: together the penta- and hexanucleotide SSRs represented 21% of the total, and 17% of the cumulative length of all imperfect SSR motifs (**Table 2**). We found that the sum of di- and trinucleotides formed the majority of perfect SSRs in all the genomes of *Solanum, Capsicum* and *Nicotiana* species searched, ranging from 67% in potato to 81% in *N. benthamiana*. However, in *Petunia* species and *Coffea canephora*, mononucleotides were the most frequent type (**Figure 1**).

**FIGURE 2:**
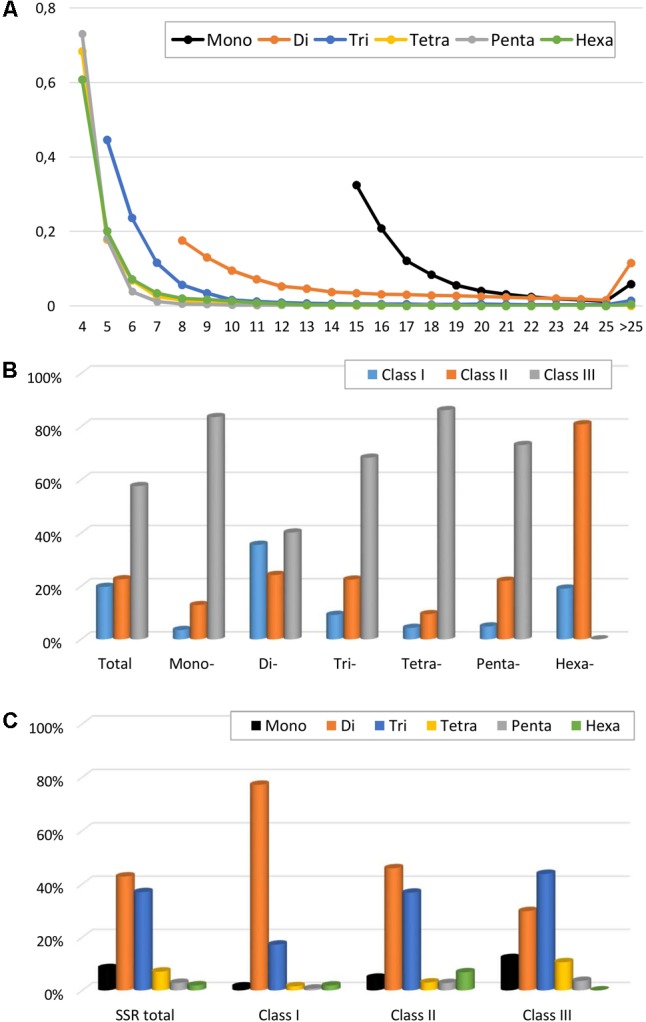
Perfect SSRs in the eggplant genome. **(A)** The relative frequency of SSR motif with different length, classified by the number of repeats. **(B)** The frequency of repeat classes (class I > 30 nt, class II 20–30 nt, class III < 20 nt. **(C)** The distribution of motif type within each class.

The variation of perfect microsatellites in the eggplant genome with regard to the number of repeat units is shown in **Figure 2** and Supplementary Table S2. In all SSR classes, it was observed that longer repeats are less abundant; a trend of decrease in SSR frequency with the increase of their repeat number has been observed in other species (Shi et al., 2013; Cheng et al., 2016; Portis et al., 2016). For example, the number of SSRs with ≤8 repeats accounted for about half the total, while those with >20 repeats accounted for less than 12%. The decrease was less marked for mononucleotides and dinucleotide motifs than for longer repeat types, with tetra- to hexanucleotide motifs showing the sharpest frequency reduction with increase of repeat (**Figure 2A**). As a consequence, the mean number of dinucleotide repeat units (15.3) was more than twice the number of trinucleotide repeat units (7.4), and about three times higher than for tetra-, penta- and hexanucleotides (5.0–5.5) (**Table 2**). Based on the length of the perfect repeat motifs, 19.7% of SSRs were considered to belong to hypervariable class I (≥30 nt). Another 22.7% were assigned as potentially variable class II (20–30 nt) types, while the remaining 57.7% represented variable class III (<20 nt) types (**Figure 2B**). Most of the mononucleotides (83.6%) belonged to class III, while for hexanucleotides classes I and II preponderated (19.1 and 80.9%, respectively) (**Figure 2B**). Class I consisted mainly of di- (77.2%) and trinucleotides (17.2%), with all the other motifs accounting for <2% (**Figure 2C**).

### Characterization of SSRs by Classified Type

Grouping of repeats into classes was carried out according to the method of Jurka and Pethiyagoda (1995). Thus the trinucleotide repeat class AAT includes (TTA)n, (TAT)n, (ATT)n, (ATA)n, and (TAA)n, which are equivalent either in different reading frames or via complementarity. A total of 336 kinds of SSR motif were found, with all the possible base combinations of mono- (only 2 types), di- (4) tri- (10) and tetranucleotide (33), plus 80 and 207 variations of penta- and hexanucleotide repeats (**Table 2**).

**FIGURE 3:**
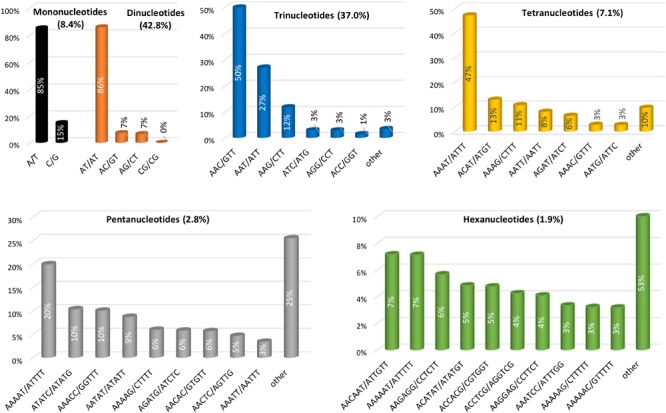
The distribution of the major repeat types in the eggplant genome.

We also evaluated in detail the individual repeat motifs for each type of SSR in the eggplant genome (**Figure 3** and Supplementary Table S3). The base composition of eggplant SSR motifs is strongly biased toward A and T; the most frequent mono- to hexa-nucleotides motifs were A/T (85.1%), AT/TA (85.9%), AAC/GTT (50.4%), AAAT/ATTT (47.1%), AAAAT/ATTTT (19.9%) and AACAAT/ATTGTT (7.1%). In terms of the distribution of different motifs, AT repeats were not only the predominant dinucleotides, but they were also the most frequent motif in the entire genome, accounting for 36.8% of the total SSRs. On the other hand, CG repeats were exceedingly rare (0.04%). Within the trinucleotide motifs, AAC, AAT, and AAG repeats were the most abundant (together representing 89%), whereas GC-rich repeats such as AGC, CCG, and ACG were uncommon.

In the same manner AT-rich tetranucleotide motifs such as AAAT, ACAT, AAAG, and AATT predominated in the eggplant genome (79% of total) while the motifs AAAAT, ATATC, AAACC, and AATAT represented nearly half (49.0%) of the total pentanucleotide repeats. Only three hexanucleotide motifs AACAAT, AAAAAT, and AAGAGG were present with a relative frequency higher than 5% (**Figure 3**). Analogous motif type distributions have been found for all the other related species analyzed here (Supplementary Table S4). It has been reported that AT-rich repeats prevail in dicot but not in monocot species, and that this difference may be explained partially by the nucleotide composition of their genomes: 34.6% GC content in dicots vs. 43.7% in monocots (Cavagnaro et al., 2010). When comparing 15 highly diverse plant species, we recently reported that among the mononucleotide motifs, A/T was predominant in all the species with the exception of rice (61.8% of C/G), while the motif AT/TA was the most frequent dimer across the genomes examined except in date palm (51.6% AG/CT). A content of CG/GC > 1% was observed only in rice, while this motif was almost absent in the other species (Portis et al., 2016).

### The Distribution of SSRs in the Chromosomes

**FIGURE 4:**
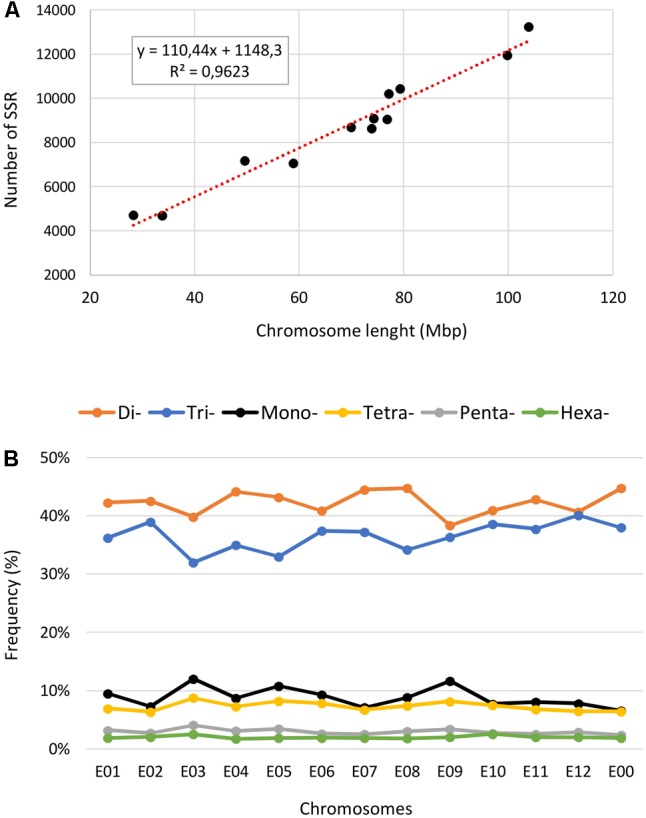
The intra-chromosomal distribution of SSRs. **(A)** Relationship between SSR number vs. chromosome length in the eggplant genome. **(B)** The frequency of mono- to hexanucleotide motifs in eggplant chromosomes.

The eggplant SSR loci identified were additionally classified according to their motif and distribution over the pseudomolecules (**Table 3** and Supplementary Table S5). About 21% of the identified SSRs were present in unanchored scaffolds (chromosome E00), while the average number detected on pseudomolecules E01–E12 was 8,746 and 11,673 for perfect and imperfect SSRs, respectively. We estimated the relationship between chromosome length and number of SSRs present on each of them and found a high correlation: *R*^2^ = 0.96, *P* < 0.01 (**Figure 4A**). The longest linkage group E01 contained the greatest number of SSRs (13,244 perfect, 17,432 imperfect, 103.9 Mbp), while the shortest (E09 and E05; 28.3 and 33.9 Mbp, respectively) hosted the fewest. However, substantial differences among chromosomes in the densities of SSRs were observed, ranging from 116.9 (E12) to 166.6 (E09) perfect SSR/Mbp (**Table 3**). On the whole a general trend of higher SSR density for short LGs (and vice versa) was observed, with the exceptions of E06 and E10 (**Table 3**). The distribution of motif types within individual chromosomes was very similar to the pattern found over the whole genome (**Figure 4B**), with di- and trinucleotide repeats the most frequent and penta-/hexanucleotides the scarcest. Mono- to trinucleotide SSRs exhibited maximum variation among LGs, with E03 and E09 showing, respectively, the lowest percentages for di- (38%) and tri- (32%) and the highest for mononucleotides (ca 12%). When considered the distribution of different motifs on a chromosome basis, the percentages of the most frequent mono- di- and tri-nucleotides were similar to those detected in the whole genome, while the relative contributions of the main tetra-, penta- and hexanucleotides varied between LGs (Supplementary Table S3).

**Table 3:** The chromosome-by-chromosome distribution of perfect, compound and imperfect SSRs.

Linkage groups	Total Mbp	Perfect								Compound		Imperfect	
		Mono-	Di-	Tri-	Tetra-	Penta-	Hexa-	Total	SSRs/ Mbp	Total	SSR/ Mbp	Total	SSRs/ Mbp
E01	103.9	1,256	5,604	4,811	916	415	242	**13,244**	127.5	**2,078**	20.0	**17,432**	167.8
E02	58.8	514	3,006	2,764	449	191	137	**7,061**	120.0	**1,223**	20.8	**9,453**	160.7
E03	70.0	1,045	3,458	2,856	760	356	212	**8,687**	124.2	**1,165**	16.7	**12,004**	171.6
E04	74.2	799	4,023	3,184	661	284	147	**9,098**	122.5	**1,271**	17.1	**12,525**	168.7
E05	33.9	508	2,027	1,522	383	163	87	**4,690**	138.6	**473**	14.0	**6,722**	198.6
E06	79.3	965	4,285	3,913	819	268	194	**10,444**	131.8	**1,874**	23.6	**13,540**	170.8
E07	99.8	847	5,330	4,461	799	297	214	**11,948**	119.7	**2,130**	21.3	**15,617**	156.4
E08	76.8	801	4,056	3,108	669	267	155	**9,056**	117.9	**1,325**	17.3	**12,356**	160.9
E09	28.3	551	1,809	1,719	381	156	91	**4,707**	166.6	**875**	31.0	**5,994**	212.1
E10	77.1	792	4,186	3,959	760	271	246	**10,214**	132.5	**1,834**	23.8	**13,465**	174.6
E11	49.6	576	3,069	2,711	489	184	139	**7,168**	144.5	**1,081**	21.8	**9,423**	189.9
E12	73.9	673	3,521	3,466	559	246	168	**8,633**	116.9	**1,471**	19.9	**11,545**	156.3
E00	233.0	1,825	12,506	10,614	1,783	652	501	**27,881**	119.7	**3,870**	16.6	**38,331**	164.5
**Total**	**1,058.6**	**11,152**	**56,880**	**49,088**	**9,428**	**3,750**	**2,533**	**132,831**	**125.5**	**20,670**	**19.5**	**178,407**	**168.5**

### Gene Context of SSRs

**FIGURE 5:**
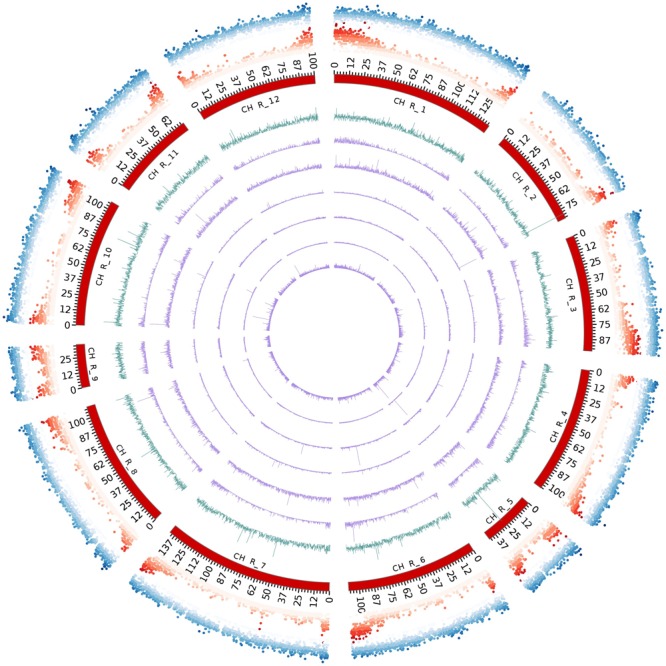
Circos diagram depicting the chromosome-scale SSR distribution (perfect). From outside to inside: repeat density, gene density, all SSRs, SSRs formed by mono-, di-, tri-, tetra-, penta- and hexanucleotides.

Using data from assembled chromosomes of the eggplant breeding line “67/3” (see footnote 2), the genomic distribution of SSRs was compared to their association with individual genes. In all, 2,449 perfect SSRs (1.84% of the total) and 3,524 imperfect SSRs (1.98%) were associated with, respectively, 2,086 and 2,924 genes (**Table 4**). This represents 6–8% of the gene space. These eggplant genes were estimated to cover a total of 132.18 Mbp (The Eggplant Genome Consortium, 2017), values which translate to a density across the gene space of 18.5 and 26.7 SSRs/Mbp for perfect and imperfect motifs, respectively. Supplementary Table S6 reports the distribution over each pseudomolecule of the identified gene SSRs classified on the basis of their repeat motif. SSRs were widely scattered on the chromosomes, except for the mono- and part of the dinucleotide motifs, which were concentrated toward the chromosome ends (**Figure 5**), similar to the gene space distribution detected in other species (The Potato Genome Sequencing Consortium, 2011; The Tomato Genome Consortium, 2012; Kim et al., 2014). Some microsatellite hotspots were observed, mostly due to very long stretches of compound microsatellites (e.g.: motifs longer than 100 repetitions) and, since they were often found between neighbor genes (e.g.: SMEL_002g164820.1 and SMEL_002g164830.1), they could be involved in gene regulation (Gao et al., 2013; Sawaya et al., 2013). Moreover, they could be exploited as putative highly polymorphic markers, since most of the highly mutable loci are compound microsatellites, comprising two or more repeated motifs (Eckert and Hile, 2009).

**Table 4:** Variation in repeat length among genic eggplant perfect and imperfect SSRs.

SSR type	Perfect motif			Imperfect motif		
	Count	%	Density (SSRs/Mbp)	Count	%	Density (SSRs/Mbp)
Mono-	215	8.8%	1.63	235	6.7%	1.78
Di-	272	11.1%	2.06	351	10.0%	2.66
Tri-	1,606	65.4%	12.15	1,755	49.8%	13.28
Tetra-	136	5.5%	1.03	247	7.0%	1.87
Penta-	32	1.3%	0.24	272	7.7%	2.06
Hexa-	188	7.7%	1.42	664	18.8%	5.02
**Total/mean**	**2,449**	**100.0%**	**18.53**	**3,524**	**100.0%**	**26.66**

**FIGURE 6:**
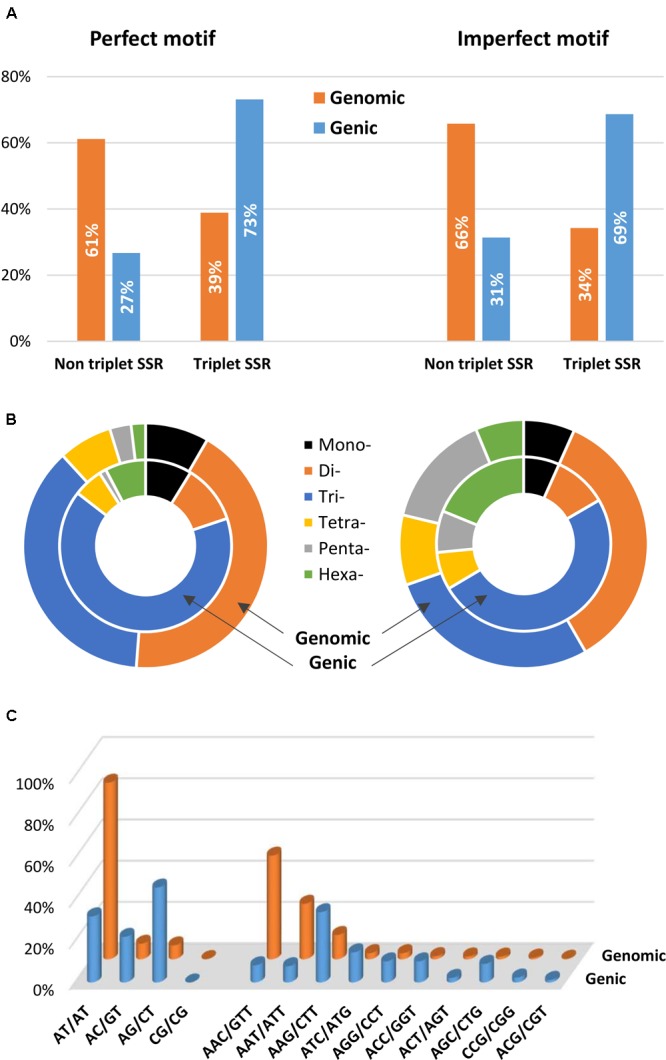
Distribution of microsatellite sizes in eggplant genome. **(A)** Non-triplet SSR vs. triplets SSR, and **(B)** distribution of repeat types within perfect and imperfect SSR motif. **(C)** Comparison between di- and trinucleotide repeats in both full genomic regions and gene space.

A comparison between SSR motifs detected in the global set of genomic and genic SSRs is reported in **Figure 6**. The total populations of SSRs across the genome and gene space were classified into triplet repeats (tri- and hexanucleotides) and non-triplet repeats (mono-, di-, tetra- and penta-nucleotides); a higher frequency in gene sequences of triplet repeats was detected for both perfect (73.1%) and imperfect (68.6%) motifs (**Figure 6A**). Trinucleotides were the most common, representing 65.4% (12.2 SSR/Mbp) and 49.8% (13.3 SSR/Mbp) of perfect and imperfect genic SSRs, respectively; the next most frequent group within perfect motifs was dinucleotides (11.1%, 2.1 SSR/Mbp), while for imperfect SSRs hexanucleotides were the second most frequent group (18.8%, 5.0 SSR/Mbp) (**Table 4** and **Figure 6B**). In analogy with other species (Toth et al., 2000; Morgante et al., 2002; Mun et al., 2006; Cavagnaro et al., 2010), even though dinucleotides are the most common repeats in the eggplant genome, tri- and hexanucleotides prevail in the gene space. This has been attributed to negative selection against frameshift mutations in coding regions; trinucleotides have increased their frequency in the coding portion as a result of mutation pressure and, possibly, positive selection for specific single amino-acid stretches (Morgante et al., 2002; Subramanian et al., 2003).

The most frequent genic SSR motif types were the trinucleotides AAG/CTT (22.2%), ATC/ATG (9.5%), AGG/CCT, and ACC/GGT. AG/CT (5.1% of the total genic SSRs) were the most common dinucleotides (**Figure 6C**). Similar patterns of motif distribution have been observed in other species. In the those compared by Morgante et al. (2002), Cavagnaro et al. (2010), and Portis et al. (2016), AT/TA repeats seem to be typical of non-transcribed regions; AG/CT prevail in gene sequences, while AC/GT and CG/GC repeats are the least frequent dinucleotides in both genomic and gene sequences. In the same way a strong bias in the distribution toward GC-rich motifs, most evident for di- tri- and hexanucleotides, was found in eggplant gene sequences. For example, trinucleotide SSRs in genes had a GC content of 43%, whereas a GC content of only 27% was found in the whole genomic trimeric SSRs.

Compared to non-coding microsatellites, genic SSR markers exhibit a higher portability among related species, boosting their utilization as anchor markers suitable for comparative genetics purposes (Varshney et al., 2005). As opposite, coding SSRs are under higher selection pressure (Toth et al., 2000), so they may not provide an adequate level of polymorphism to distinguish between closely related ecotypes/varieties. Indeed, eggplant genic SSRs provide a reduced set of potentially variable SSRs, as their repeat numbers are lower than in the whole genome, with 84.2% of SSRs containing ten or fewer repeats and only 3.3% having 20 or more repeats. However, genic SSRs represent a relevant class of ‘functional markers’ as their presence in transcripts has been shown to play a critical role in gene expression/function in both humans (i.e.: microsatellite instability, MSI; Brouwer et al., 2009; Nelson et al., 2013) and plants, where MSI is known to increase with plant development in Arabidopsis (Golubov et al., 2010). With this aim, we compared the set of SSR-containing genes in the reference eggplant gene space, assessing the specific gene regulation functions which are frequently present.

**FIGURE 7:**
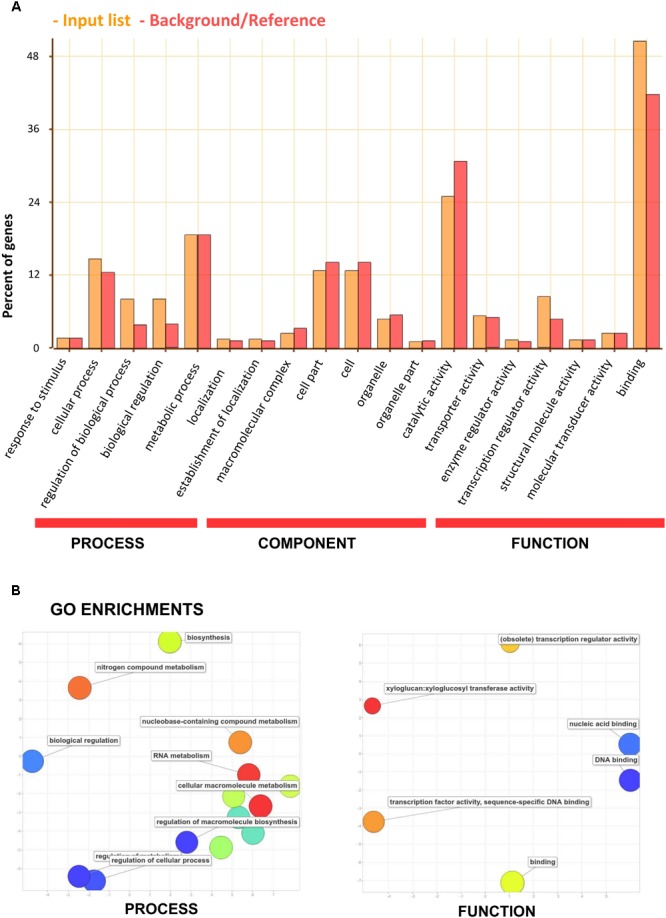
Functional analysis (gene ontology) of the set of eggplant genes containing SSRs. **(A)** GO categorization. The orange bars indicate input genes, while the red bars indicate background genes. **(B)** REVIGO summary of “biological process” and “molecular function” enriched terms. The bubble size is proportional to the log_10_ (*p*-value) of enrichment analyses and its color is also a function of log_10_ (*p*-value) of enrichment analyses (blue: low, -red: high *p*-value). The *x* and *y*-axes reflect semantic similarity according to the REVIGO algorithm (similar GO terms appear close together).

The genes containing one or more SSRs were found within 38 sub-GO categories of three main GO categories [“biological processes” (BP), “molecular functions” (MF) and “cellular components” (CC)]. Over-representation was found for a number of gene families (**Figure 7** and Supplementary Table S7); for BP in the sub-categories “regulation of gene expression” (GO:0010468), “regulation of transcription” (GO:0045449), and “transcription” (GO:0006350); for MF, “nucleic acid binding” (GO:0003676) and “transcription regulator activity” (GO:0030528). No enrichment was observed for CC. The occurrence of SSRs within specific gene functions has previously been observed (Yu et al., 2010; Kujur et al., 2013; Liu et al., 2015; Portis et al., 2016; Scaglione et al., 2016) and transcription factors form a significant class of genes containing SSRs. Moreover, the critical role of transcription factors carrying microsatellites has been noted (Li et al., 2004), and this requires clarification in relation to species diversification within Solanaceae.

### EgMiDB System Architecture, Features and Utility

**FIGURE 8:**
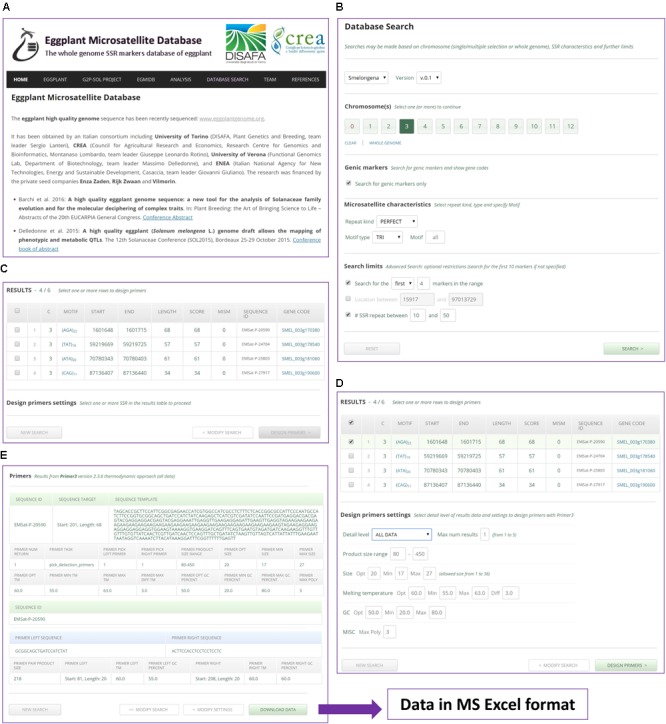
A worked example of an SSR search and primer design using *EgMiDB*. **(A)** *EgMiDB* Home page. **(B)** Settings given for chromosome selection and SSR search. **(C)** The SSR output. **(D)** Settings given for the design of primers. **(E)** Suggested primers and the downloading of the result.

The public domain *EgMiDB* available at www.eggplantmicrosatellite.org provides a searchable interface to the microsatellite data reported in this study. It offers similar features to the *CyMSatDB* database ^9^ (Portis et al., 2016). In brief, it can be used to retrieve SSRs based on a wide range of simple and complex searches, with single or chained queries. More than one chromosome may be selected. Researches can limit the search via location and number of markers in the required range. The output lists a wide range of all necessary information, including an optional download of the flanking sequences. A ‘Design Primers’ button provides detailed data including sequences, melting temperatures, etc., with the facility to download in Excel format (**Figure 8**).

*EgMiDB* is highly user-friendly, optimized to be accessed on all mobile devices. It implements input checking with auto-correction of errors. It: (i) displays default values or ranges; (ii) auto-corrects data immediately on entry rather than when a query is run; (iii) provides comprehensible error messages; and (iv) indicates when a field is valid. It also provides a simple interface for use by administrators when importing data into the system, and facilitates conversion of SciRoKo output into data formatted for EgMiDB.

With the marker information present in *EgMiDB*, the standard linkage map’s marker density can be increased, and this will contribute to QTL and gene mapping studies. In order to facilitate the use of these markers, we have implemented several plugins to generate primers at user-defined chromosomal locations, which can be directly exported for use in genotyping assays. Additionally, the developed SSR markers can also be used in DUS testing for variety identification in multiplex mode. From our large marker dataset, the identification of markers with thermodynamic compatibility for multiplex designing can be also accomplished.

### Marker Validation

**FIGURE 9:**
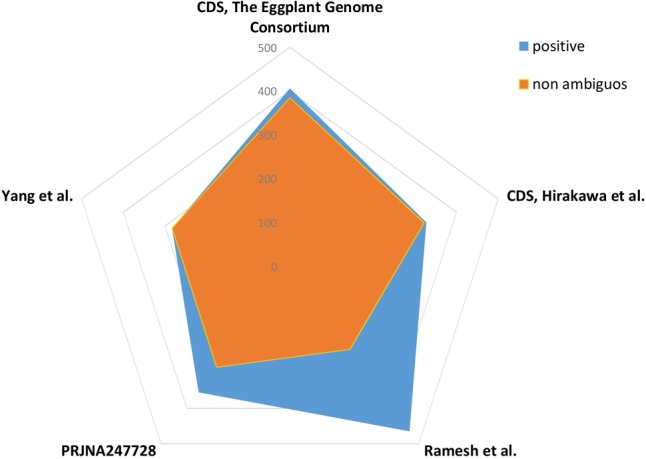
Simple sequence repeat *in silico* validation. Relative number of microsatellites showing positive signal following *in silico* validation of 1000 randomly selected loci in three transcriptomes (Yang et al., 2014; Ramesh et al., 2016; PRJNA247728, unpublished) and two CDS sets (from eggplant genomic projects; Hirakawa et al., 2014; The Eggplant Genome Consortium, 2017).

In addition to understanding the characteristics of SSR distribution, the development of molecular markers for eggplant based on these loci was one of the most important objectives in this study. An *in silico* validation exercise was carried out, taking advantage of available transcriptomic/genomic resources. Normal methods for validating SSR markers involve employing them to prime PCRs containing appropriate template DNA. Here, *in silico* validation was possible because of the sequence data available from five eggplant accessions. The CDS set derived from the inbred “67/3” line (The Eggplant Genome Consortium, 2017) provided validation of over one-third (385/999) of the markers, while the CDS set from cv. “Nakate-Shinkuro” (Hirakawa et al., 2014) validated a slightly lower proportion (322/999). The other three assembled transcriptomes gave from 233/464 to 283/283 non-ambiguous *in silico* PCR signals (**Figure 9**). By taking into account ambiguous amplification, validation percentages were substantially increased in fragmented sets (i.e., transcriptomes).

By using the genes predicted from the two sequenced eggplant varieties, almost 32–39% of primer pairs were predicted to be functional. When data from the three different assembled transcriptomes was included, the validation level was reduced to 23–28%, which can be considered satisfactory, since in previous similar studies validation success rates ranging from 50% (Portis et al., 2016) to 11% (Iquebal et al., 2013) have been reported. The presence of non-validated markers may be attributed to the presence of a large intron(s) between the pair of primer sites or the presence of primers in intron regions, because the sequences used for primer design are genomic-based. All the 999 validated SSRs were loci on genes, but only 345 (35%) of these genes were mono-exonic, a percentage close to the success rate found in the validation tests. Differences in validation between cultivar-derived datasets will also result from the genetic structure of the varieties used to assemble/predict genes. Even taking this into account, our first *EgMiDB* provides key information for genetics and breeding studies as well as the integration of un-mapped scaffolds for improvement and optimization of the eggplant genome sequence.

## Conclusion

The main goal of the present study was to identify a large set of valid SSR markers in eggplant, by adopting key standards including: (i) an SSR length of not less than 15 bp and (ii) a minimum of four repeat units per SSR. These parameters were chosen since microsatellite polymorphisms and mutation rates correlate positively with increase in the number of repeat units (Weber, 1990) as this promotes higher rates of strand-slippage during replication (Whittaker et al., 2003). A total of 133k/178k eggplant-specific perfect/imperfect microsatellite loci are reported here, which were mined using a whole genome bioinformatics survey. A large set of long/hypervariable and potentially variable SSRs has been identified. Because microsatellites are, as expected, ubiquitously distributed, we detected higher SSR repeat content for longer chromosomes (Zietkiewicz et al., 1994) as well as homogeneous distribution of SSRs. These inherent attributes of microsatellites make them desirable markers.

The database *EgMiDB* contains all the available information related to genomic and genic perfect/imperfect microsatellite loci, with unrestricted public access. Through an easy-to-use web interface and a highly customized primer-designing tool, it is optimized to cater for the needs of plant biologists and breeders. It is very flexible and allows access with user-defined options. The database will help in selection of microsatellites of a particular type and also in identification of all putative microsatellite sites within specific genomic regions, such as functional microsatellites present in gene sequences. Furthermore, it makes it possible the identification of markers with thermodynamic compatibility for multiplex designing by allowing a large number of samples to be screened in a given experiment. EgMiDB will thus contribute to uniform bioinformatics workflow strategy to achieve SSR genotyping tailored to eggplant, and represents a key tool for making progress in both basic genomics research and molecular breeding of the species. The database will be constantly updated as additional sequencing data for eggplant genotypes become available, allowing rapid and simple *in silico* identification of SSRs including those polymorphic.

## Author Contributions

EP, SL, FP, GLR, and AA conceived and designed the research. EP, LB, LT, and AA analyzed the data. FP and LV designed and constructed the web database. EP and AA drafted the manuscript. SL, LT, and GLR reviewed and edited the manuscript. All authors read and approved the manuscript.

## Conflict of Interest Statement

The authors declare that the research was conducted in the absence of any commercial or financial relationships that could be construed as a potential conflict of interest. The handling Editor declared a past co-authorship with several of the authors EP, SL, LB, AA.

## Footnotes

1 http://faostat3.fao.org/

2 www.eggplantgenome.org

3 www.eggplantmicrosatellite.org/

4 http://kofler.or.at/bioinformatics/SciRoKo

5 http://bedtools.readthedocs.io

6 http://systemsbiology.cau.edu.cn/agriGOv2/

7 revigo.irb.hr/

8 https://sourceforge.net/projects/primer3

9 www.artichokegenome.unito.it/cymsatdb/

## References

[B1] Acquadro A. Barchi L. Gramazio P. Portis E. Vilanova S. Comino C. (2017). Coding SNPs analysis highlights genetic relationships and evolution pattern in eggplant complexes. *PLoS One* 12:e0180774. 10.1371/journal.pone.0180774 28686642 PMC5501601

[B2] Barchi L. Lanteri S. Portis E. Acquadro A. Valè G. Toppino L. (2011). Identification of SNP and SSR markers in eggplant using RAD tag sequencing. *BMC Genomics* 12:304. 10.1186/1471-2164-12-304 21663628 PMC3128069

[B3] Barchi L. Lanteri S. Portis E. Stagel A. Valè G. Toppino L. (2010). Segregation distortion and linkage analysis in eggplant (*Solanum melongena* L.). *Genome* 53 805–815. 10.1139/G10-073 20962887

[B4] Barchi L. Lanteri S. Portis E. Valè G. Volante A. Pulcini L. (2012). A RAD Tag derived marker based eggplant linkage map and the location of QTLs determining anthocyanin pigmentation. *PLoS One* 7:e43740. 10.1371/journal.pone.0043740 22912903 PMC3422253

[B5] Behura S. K. Severson D. W. (2014). Motif mismatches in microsatellites: insights from genome-wide investigation among 20 insect species. *DNA Res.* 22 29–38. 10.1093/dnares/dsu036 25378245 PMC4379975

[B6] Brouwer J. R. Willemsen R. Oostra B. A. (2009). Microsatellite repeat instability and neurological disease. *BioEssays* 31 71–83. 10.1002/bies.080122 19154005 PMC4321794

[B7] Cavagnaro P. F. Senalik D. A. Yang L. Simon P. W. Harkins T. T. Kodira C. D. (2010). Genome-wide characterization of simple sequence repeats in cucumber (*Cucumis sativus* L.). *BMC Genomics* 11:569. 10.1186/1471-2164-11-569 20950470 PMC3091718

[B8] Cericola F. Portis E. Toppino L. Barchi L. Acciarri N. Ciriaci T. (2013). The population structure and diversity of eggplant from Asia and the Mediterranean Basin. *PLoS One* 8:e73702. 10.1371/journal.pone.0073702 24040032 PMC3765357

[B9] Cheng J. Zhao Z. Li B. Qin C. Wu Z. Trejo-Saavedra D. L. (2016). A comprehensive characterization of simple sequence repeats in pepper genomes provides valuable resources for marker development in Capsicum. *Sci. Rep.* 6:18919. 10.1038/srep18919 26739748 PMC4703971

[B10] Eckert K. A. Hile S. E. (2009). Every microsatellite is different: intrinsic DNA features dictate mutagenesis of common microsatellites present in the human genome. *Mol. Carcinog.* 48 379–388. 10.1002/mc.20499 19306292 PMC2731485

[B11] Fukuoka H. Miyatake K. Nunome T. Negoro S. Shirasawa K. Isobe S. (2012). Development of gene-based markers and construction of an integrated linkage map in eggplant by using *Solanum* orthologous (SOL) gene sets. *Theor. Appl. Genet.* 125 47–56. 10.1007/s00122-012-1815-9 22350090

[B12] Gao C. Ren X. Mason A. S. Li J. Wang W. Xiao M. (2013). Revisiting an important component of plant genomes: microsatellites. *Funct. Plant Biol.* 40 645–661. 10.1071/FP12325 32481138

[B13] Golubov A. Yao Y. Maheshwari P. Bilichak A. Boyko A. Belzile F. (2010). Microsatellite instability in Arabidopsis increases with plant development. *Plant Physiol.* 154 1415–1427. 10.1104/pp.110.162933 20817752 PMC2971617

[B14] Gramazio P. Blanca J. Ziarsolo P. Herraiz F. J. Plazas M. Prohens J. (2016). Transcriptome analysis and molecular marker discovery in *Solanum incanum* and *S. aethiopicum*, two close relatives of the common eggplant (*Solanum melongena*) with interest for breeding. *BMC Genomics* 17:300. 10.1186/s12864-016-2631-4 27108408 PMC4841963

[B15] Gramazio P. Prohens J. Borras D. Plazas M. Herraiz F. J. Vilanova S. (2017). Comparison of transcriptome-derived simple sequence repeat (SSR) and single nucleotide polymorphism (SNP) markers for genetic fingerprinting, diversity evaluation, and establishment of relationships in eggplants. *Euphytica* 213:264 10.1007/s10681-017-2057-3

[B16] Hirakawa H. Shirasawa K. Miyatake K. Nunome T. Negoro S. Ohyama A. (2014). Draft genome sequence of eggplant (*Solanum melongena* L.): the representative solanum species indigenous to the old world. *DNA Res.* 21 649–660. 10.1093/dnares/dsu027 25233906 PMC4263298

[B17] Hurtado M. Vilanova S. Plazas M. Gramazio P. Fonseka H. H. Fonseka R. (2012). Diversity and relationships of eggplants from three geographically distant secondary centers of diversity. *PLoS One* 7:e41748. 10.1371/journal.pone.0041748 22848589 PMC3407184

[B18] Iquebal M. A. Sarika J. Arora V. Verma N. Rai A. Kumar D. (2013). First whole genome based microsatellite DNA marker database of tomato for mapping and variety identification. *BMC Plant Biol.* 13:197 10.1186/1471-2229-13-197

[B19] Jurka J. Pethiyagoda C. (1995). Simple repetitive DNA sequences from primates: compilation and analysis. *J. Mol. Evol.* 40 120–126. 10.1007/BF00167107 7699718

[B20] Kim S. Park M. Yeom S.-I. Kim Y.-M. Lee J. M. Lee H.-A. (2014). Genome sequence of the hot pepper provides insights into the evolution of pungency in Capsicum species. *Nat. Genet.* 46 270–278. 10.1038/ng.2877 24441736

[B21] Kujur A. Bajaj D. Saxena M. S. Tripathi S. Upadhyaya H. D. Gowda C. L. (2013). Functionally relevant microsatellite markers from chickpea transcription factor genes for efficient genotyping applications and trait association mapping. *DNA Res.* 20 355–374. 10.1093/dnares/dst015 23633531 PMC3738162

[B22] Li Y. C. Korol A. B. Fahima T. Nevo E. (2004). Microsatellites within genes: structure, function, and evolution. *Mol. Biol. Evol.* 21 991–1007. 10.1093/molbev/msh073 14963101

[B23] Liu W. Jia X. Liu Z. Zhang Z. Wang Y. Liu Z. (2015). Development and characterization of transcription factor gene-derived microsatellite (TFGM) markers in *Medicago truncatula* and their transferability in leguminous and non-leguminous species. *Molecules* 20 8759–8771. 10.3390/molecules20058759 25988608 PMC6272326

[B24] Meyer R. S. Karol K. G. Little D. P. Nee M. H. Litt A. (2012). Phylogeographic relationships among Asian eggplants and new perspectives on eggplant domestication. *Mol. Phylogenet. Evol.* 63 685–701. 10.1016/j.ympev.2012.02.006 22387533

[B25] Morgante M. Hanafey M. Powell W. (2002). Microsatellites are preferentially associated with nonrepetitive DNA in plant genomes. *Nat. Genet.* 30 194–200. 10.1038/ng822 11799393

[B26] Mun J. H. Kim D. J. Choi H. K. Gish J. Debellé F. Mudge J. (2006). Distribution of microsatellites in the genome of *Medicago truncatula*: a resource of genetic markers that integrate genetic and physical maps. *Genetics* 172 2541–2555. 10.1534/genetics.105.054791 16489220 PMC1456377

[B27] Munoz-Falcon J. E. Vilanova S. Plazas M. Prohens J. (2011). Diversity, relationships and genetic fingerprinting of the Listada de Gandía eggplant landrace using genomic SSRs and EST-SSRs. *Sci. Hortic.* 129 238–246. 10.1016/j.scienta.2011.03.034

[B28] Nelson D. L. Orr H. T. Warren S. T. (2013). The unstable repeats - three evolving faces of neurological disease. *Neuron* 77 825–843. 10.1016/j.neuron.2013.02.022 23473314 PMC3608403

[B29] Nunome T. Negoro S. Kono I. Kanamori H. Miyatake K. Yamaguchi H. (2009). Development of SSR markers derived from SSR-enriched genomic library of eggplant (Solanum melongena L.). *Theor. Appl. Genet.* 119 1143–1153. 10.1007/s00122-009-1116-0 19662343

[B30] Nunome T. Suwabe K. Ohyama A. Fukuoka H. (2003b). Characterization of trinucleotide microsatellites in Eggplant. *Breed. Sci.* 53 77–83. 10.1270/jsbbs.53.77

[B31] Nunome T. Suwabe K. Iketani H. Hirai M. (2003a). Identification and characterization of microsatellites in eggplant. *Plant Breed.* 122 256–262. 10.1046/j.1439-0523.2003.00816.x

[B32] Portis E. Portis F. Valente L. Moglia A. Barchi L. Lanteri S. (2016). A genome-wide survey of the microsatellite content of the globe artichoke genome and the development of a web-based database. *PLoS One* 11:e0162841. 10.1371/journal.pone.0162841 27648830 PMC5029897

[B33] Prohens J. Plazas M. Raigon M. D. Seguì-Simarro J. Stommel J. Vilanova S. (2012). Characterization of interspecific hybrids and first backcross generations from crosses between two cultivated eggplants (*Solanum melongena* and S. *aethiopicum* Kumba group) and implications for eggplant breeding. *Euphytica* 186 517–538. 10.1007/s10681-012-0652-x

[B34] Ramesh K. R. Hemalatha R. Vijayendra C. A. Arshi U. Z. Dushyant S. B. Dinesh K. B. (2016). Transcriptome analysis of *Solanum melongena* L. (eggplant) fruit to identify putative allergens and their epitopes. *Gene* 576 64–71. 10.1016/j.gene.2015.09.064 26424595

[B35] Sawaya S. Bagshaw A. Buschiazzo E. Kumar P. Chowdhury S. Black M. A. (2013). Microsatellite tandem repeats are abundant in human promoters and are associated with regulatory elements. *PLoS One* 8:e54710. 10.1371/journal.pone.0054710 23405090 PMC3566118

[B36] Scaglione D. Reyes-Chin-Wo S. Acquadro A. Froenicke L. Portis E. Beitel C. (2016). The genome sequence of the outbreeding globe artichoke constructed de novo incorporating a phase-aware low-pass sequencing strategy of F1 progeny. *Sci. Rep.* 6:19427. 10.1038/srep19427 26786968 PMC4726258

[B37] Shi J. Huang S. Fu D. Yu J. Wang X. Hua W. (2013). Evolutionary dynamics of microsatellite distribution in plants: insight from the comparison of sequenced *Brassica, Arabidopsis* and other angiosperm species. *PLoS One* 8:e59988. 10.1371/journal.pone.0059988 23555856 PMC3610691

[B38] Stàgel A. Portis E. Toppino L. Rotino G. L. Lanteri S. (2008). Gene-based microsatellite development for mapping and phylogeny studies in eggplant. *BMC Genomics* 9:357. 10.1186/1471-2164-9-357 18667065 PMC2527019

[B39] Subramanian S. Mishra R. K. Singh L. (2003). Genome-wide analysis of microsatellite repeats in humans: their abundance and density in specific genomic regions. *Genome Biol.* 4:R13. 10.1186/gb-2003-4-2-r13 12620123 PMC151303

[B40] The Eggplant Genome Consortium (2017). *The Eggplant Genome Reveals Key Events in Solanaceae Evolution.* Available at: https://pag.confex.com/pag/xxv/meetingapp.cgi/Paper/23484

[B41] The Potato Genome Sequencing Consortium (2011). Genome sequence and analysis of the tuber crop potato. *Nature* 475 189–195. 10.1038/nature10158 21743474

[B42] The Tomato Genome Consortium (2012). The tomato genome sequence provides insights into fleshy fruit evolution. *Nature* 485 635–641. 10.1038/nature11119 22660326 PMC3378239

[B43] Toth G. Gaspari Z. Jurka J. (2000). Microsatellites in different eukaryotic genomes: survey and analysis. *Genome Res.* 10 967–981. 10.1101/gr.10.7.967 10899146 PMC310925

[B44] Varshney R. K. Graner A. Sorrells M. E. (2005). Genic microsatellite markers in plants: features and applications. *Trend Biotechnol.* 23 48–55. 10.1016/j.tibtech.2004.11.005 15629858

[B45] Vilanova S. Manzur J. Prohens J. (2012). Development and characterization of genomic simple sequence repeat markers in eggplant and their application to the study of diversity and relationships in a collection of different cultivar types and origins. *Mol. Breed.* 30 647–660. 10.1007/s11032-011-9650-2

[B46] Weber J. L. (1990). Informativeness of human (dC-dA)n.(dG-dT)n polymorphisms. *Genomics* 7 524–530. 10.1016/0888-7543(90)90195-Z 1974878

[B47] Whittaker J. C. Harbord R. M. Boxall N. Mackay I. Dawson G. Sibly R. M. (2003). Likelihood-based estimation of microsatellite mutation rates. *Genetics* 164 781–787. 12807796 10.1093/genetics/164.2.781 PMC1462577

[B48] Xu J. Liu L. Xu Y. Chen C. Rong T. Ali F. (2013). Development and characterization of simple sequence repeat markers providing genome-wide coverage and high resolution in maize. *DNA Res.* 20 497–509. 10.1093/dnares/dst026 23804557 PMC3789560

[B49] Yang X. Cheng Y. F. Deng C. Ma Y. Wang Z. W. Chen X. H. (2014). Comparative transcriptome analysis of eggplant (*Solanum melongena* L.) and turkey berry (*Solanum torvum* Sw.): phylogenomics and disease resistance analysis. *BMC Genomics* 15:412. 10.1186/1471-2164-15-412 24885385 PMC4070557

[B50] Yu J. K. Paik H. Choi J. P. Han J. H. Choe J. K. Hur C. G. (2010). Functional domain marker (FDM): an in silico demonstration in Solanaceae using simple sequence repeats (SSRs). *Plant Mol. Biol. Rep.* 28 352–356. 10.1007/s11105-009-0154-8

[B51] Zhao X. Tian Y. Yang R. Feng H. Ouyang Q. Tian Y. (2012). Coevolution between simple sequence repeats (SSRs) and virus genome size. *BMC Genomics* 13:435. 10.1186/1471-2164-13-435 22931422 PMC3585866

[B52] Zietkiewicz E. Rafalski A. Labuda D. (1994). Genome finger printing by simple sequence repeat (SSR)-anchored polymerase chain reaction amplification. *Genomics* 20 176–218. 10.1006/geno.1994.1151 8020964

